# High PYGL Expression Predicts Poor Prognosis in Human Gliomas

**DOI:** 10.3389/fneur.2021.652931

**Published:** 2021-06-11

**Authors:** Chang-yi Zhao, Chun-hui Hua, Chang-hua Li, Rui-zhe Zheng, Xin-yuan Li

**Affiliations:** Department of Neurosurgery, Tongren Hospital, Shanghai Jiao Tong University School of Medicine, Shanghai, China

**Keywords:** PYGL, prognosis, TAMs, gliomas, expression

## Abstract

**Background:** PYGL has been reported as a glycogen degradation-related gene, which is up-regulated in many tumors. This study was designed to investigate the predictive value of high PYGL expression in patients with gliomas through bioinformatics analysis of the gene transcriptome and the single-cell sequencing data.

**Methods:** The gene transcriptome data of 595 glioma patients from the TCGA database and the single-cell RNA sequencing data of 7,930 GBM cells from the GEO database were included in the study. Differential analysis was used to find the distribution of expression of PYGL in different groups of glioma patients. OS analysis was used to assess the influence of the high expression of PYGL on the prognosis of patients. The reliability of its prediction was evaluated by the AUC of ROC and the C-index. The GSEA be used to reveal potential mechanisms. The single-cell analysis was used to observe the high expression of PYGL in different cell groups to further analyze the mechanism of its prediction.

**Results:** Differential analysis identified the expression level of PYGL is positively associated with glioma malignancy. OS analysis and Cox regression analyses showed high expression of PYGL was an independent factor for poor prognosis of gliomas (*p* < 0.05). The AUC values were 0.838 (1-year ROC), 0.864 (3-year ROC) and 0.833 (5-year ROC). The C index was 0.81. The GSEA showed that gene sets related to MTORC1 signaling, glycolysis, hypoxia, PI3K/AKT/mTOR signaling, KRAS signaling up and angiogenesis were differentially enriched in the high PYGL expression phenotype. The single-cell sequencing data analysis showed TAMs and malignant cells in GBM tissues expressed a high level of PYGL.

**Conclusion:** The high expression of PYGL is an independent predictor of poor prognosis in patients with glioma.

## Introduction

Glioma is the most common primary tumor in the brain and it is also one of the most invasive tumors in human beings. In the past, it has been widely accepted that the median survival of patients with gliomas depends largely on the histological grade of the tumor, isocitrate dehydrogenase (IDH) mutation status, O6-methylguanine-DNA methyl-transferase (MGMT) gene promoter methylation status, and 1p/19q codeletion (1–3).

Recently, the modulated glycogen metabolism of tumors becomes a promising focus of attention. During the metabolic adaptation and reprogramming progress, breakdown and synthesis of glycogen involve in the pathological activity of the numerous metabolic pathways. Glycogen phosphorylase (GP), the key enzyme of glycogenolysis, is responsible for the release of glucose-1-phosphate (G1P) from hepatic and muscle glycogen under physiological conditions. So far, there have three GP isoforms been found in the normal human body: PYGM (muscle), PYGL (liver), and PYGB (brain) (4). Previous studies have demonstrated that the turnover of glycogen is altered in various tumor cells. To our best knowledge, PYGL is one of the gene related to hypoxia metabolism and was found to be up-regulated in head and neck squamous cell carcinomas (HNSCCs) and breast cancers (5). It was suggested that the glycogen degradation regulated by PYGL could maintain the proliferation of cancer cells (6). However, there have been no previous studies to explore the relationship between the expression of PYGL and the prognosis of patients with gliomas. In addition, there must be a key step to elongate glycogen branches prior to glycogen degradation, among which glycogen synthase (GS) catalyzed such an essential metabolic pathway. GS has two isoforms: GYS1 (muscle) and GYS2 (liver) (7). In the past, GYS1 induced glycogen accumulation and promoted tumor progression under hypoxia conditions have also been found in many cancer cell lines (8, 9). Concordantly, it is not clear that the pathophysiological mechanism of GYS1 in regulating gliomas.

In the present study, we provide evidence that high PYGL expression plays an independent role in predicting the poor prognosis of glioma patients. We demonstrated that tumor-associated macrophages (TAMs) in GBM tissues expressed a high level of PYGL. In addition, we further explored the underlying pathophysiologic mechanisms of abnormal glycogen metabolism in human gliomas.

## Materials and Methods

### Raw Data

Transcriptome RNA-seq data of 677 glioma cases (low-grade glioma samples, 511 cases; glioblastoma samples, 166 cases) and the corresponding clinical data were downloaded from The Cancer Genome Atlas (TCGA) database. Eighty-two patients with incomplete clinical data or survival time <30 days were excluded from this analysis, and then 595 patients were included in the study. The single-cell RNA sequencing data are publicly available from the Gene Expression Omnibus (GEO) database, accession number GSE131928 (7,930 cells from 28 patients with GBM).

### Statistical Analysis

#### PYGL Differential Expression Analysis

World Health Organization (WHO) grade II, III, and IV gliomas from the TCGA database were included in the analysis. The mRNA expression of PYGL was compared by groups. Patients were divided respectively into three different age categories (<40/40–59/≥60 years), two sex categories (male/female), three different malignant categories (WHO grade II/ WHO III/ WHO IV), and two IDH mutation status groups (IDH-mutated/ IDH-wild-type). Kruskal-Wallis test with Dunn *post hoc* tests and Wilcoxon test were adopted to analyze the differential expression between groups. The statistical analysis was performed using R software 3.6.3 (https://www.r-project.org/).

#### Survival Analysis

Survival analysis was used to compare the overall survival (OS) in 595 glioma patients, the survival curve was generated by the R survival package or the survminer package. A statistically significant value was set at *P* < 0.05. Kaplan-Meier survival analysis was performed on all patients, different malignant degrees, different age groups, and different IDH status, and their median expression values were used as the thresholds of survival analysis. Univariate and multivariate Cox hazards regression analyses were used to determine whether PYGL can be an independent prognostic factor. ROC curve was used to analyze the predictive value of PYGL, and the predicting ability is considered acceptable when the area under the curve (AUC) is higher than 0.7. Predictive accuracy was determined using Harrell's concordance index (C-index), which was generated by the R survival package.

#### Gene Set Enrichment Analysis (GSEA)

To reveal potential mechanisms underlying the effect of PYGL expression on glioma prognosis, Gene Set Enrichment Analysis (GSEA) was performed to detect functional categories enriched in the high and low PYGL expression groups. Gene sets with a normal *P*-value < 0.05 and false discovery rate (FDR) *q*-value < 0.25 were considered to be significantly enriched. GSEA was performed using GSEA 4.0.3 software (http://www.broadinstitute.org/gsea).

#### Analysis of Single-Cell mRNA Sequencing

The single-cell RNA sequencing data were processed by normalization, PCA and TSNE dimensionality reduction, and clustering using the Seurat R package version 3.1.5. Then clusters were combined according to cell types. Finally, violin plots, scatter plots, and bubble plots were used to show the distribution of cells with high expression of PYGL and GYS1 in different cell types.

## Results

### Clinical Characteristics of the Study Population

The clinical data of 595 patients were downloaded from the TCGA database, including patients' age, gender, pathologic type, chemotherapy, radiotherapy, and survival status (Table 1).

**Table 1:** Clinical characteristics of the study population.

**Characteristic**	**N (%)**
Age	
<40 years	218 (36.64)
40–59 years	240 (40.34)
≥60 years	137 (23.03)
Gender	
Male	337 (56.64)
Female	258 (43.36)
Malignant grade	
WHO II	271 (45.55)
WHO III	181 (30.42)
WHO IV	143 (24.03)
IDH	
IDH mutated	321 (53.95)
IDH wild type	274 (46.05)
Chemotherapy	
Yes	411 (69.08)
No	184 (30.92)
Radiotherapy	
Yes	433 (72.77)
No	162 (27.23)
Survival status	
Death	232 (38.99)
Survival	363 (61.01)

### Positive Correlation Between High Expression of PYGL and Glioma Malignancy

**Figure 1:**
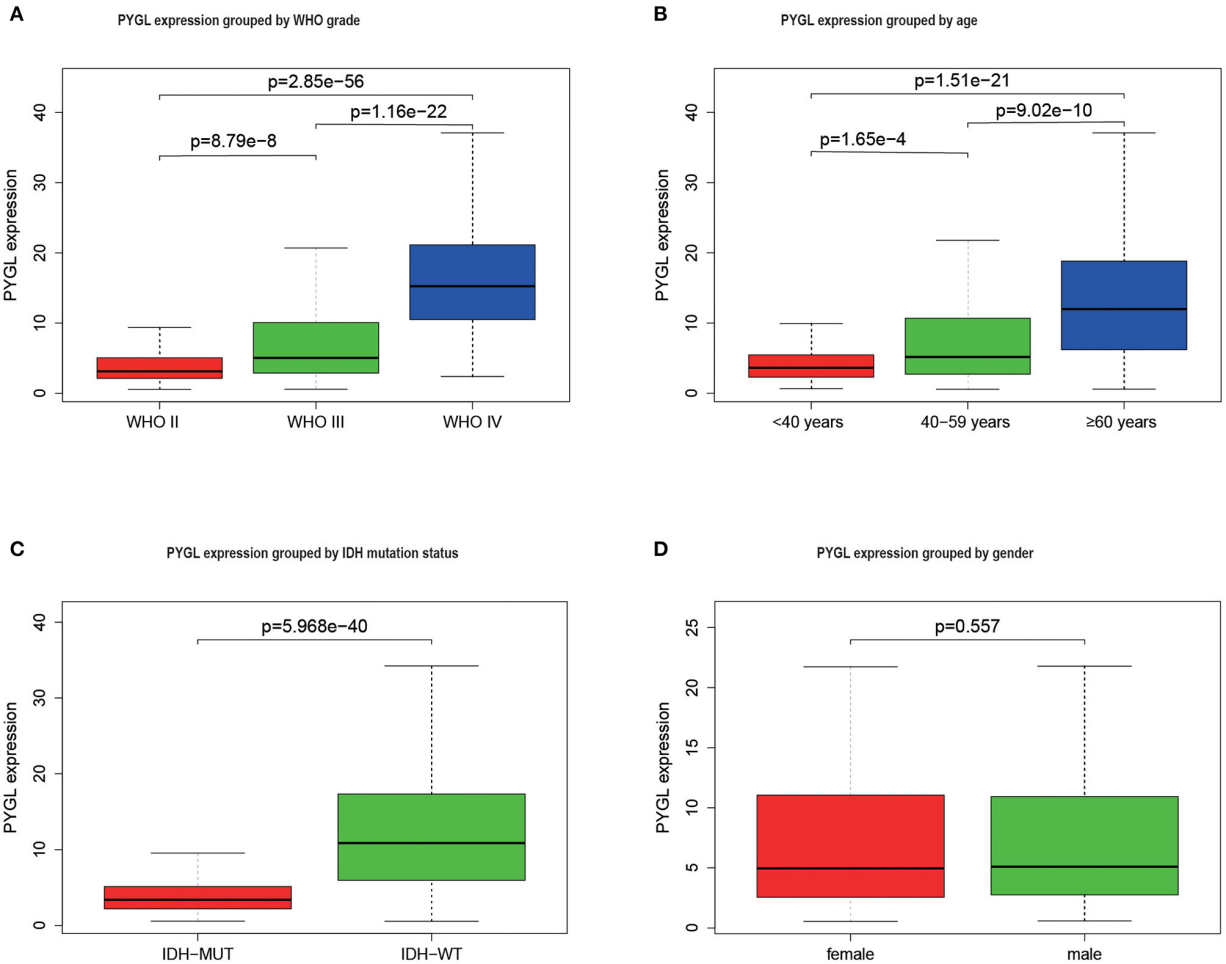
PYGL expression in glioma. **(A)** PYGL expression at different malignant grade categories. **(B)** PYGL expression at different age categories. **(C)** PYGL expression at two IDH mutation status groups. **(D)** PYGL expression at two sex categories.

The mRNA expressions of PYGL in gliomas among groups with different clinical characteristics were compared using the Kruskal–Wallis test or Wilcoxon test. The results showed that PYGL expression was significantly higher in the WHO IV grade glioma tissues than that in WHO II grade group (*P* = 2.85e-56) and WHO III grade group (*P* = 1.16e-22; Figure 1A). Moreover, the comparison between different age groups and expression of PYGL showed a remarkable positive correlation (Figure 1B). The expression of PYGL in the IDH-wild-type group was significantly higher than that in the IDH-mutated group (*P* = 5.97e-40; Figure 1C). There was no significant difference between males and females (*p* = 0.557; Figure 1D). The correlation analysis between malignant grade of glioma and age performed using Kendall tau rank test shows that their correlation is not very high (tau = 0.3496482, *p*-value <2.2e-16). These results suggest that the expression level of PYGL is positively associated with glioma malignancy.

### High PYGL Expression Predicted Poor Prognosis

**Figure 2:**
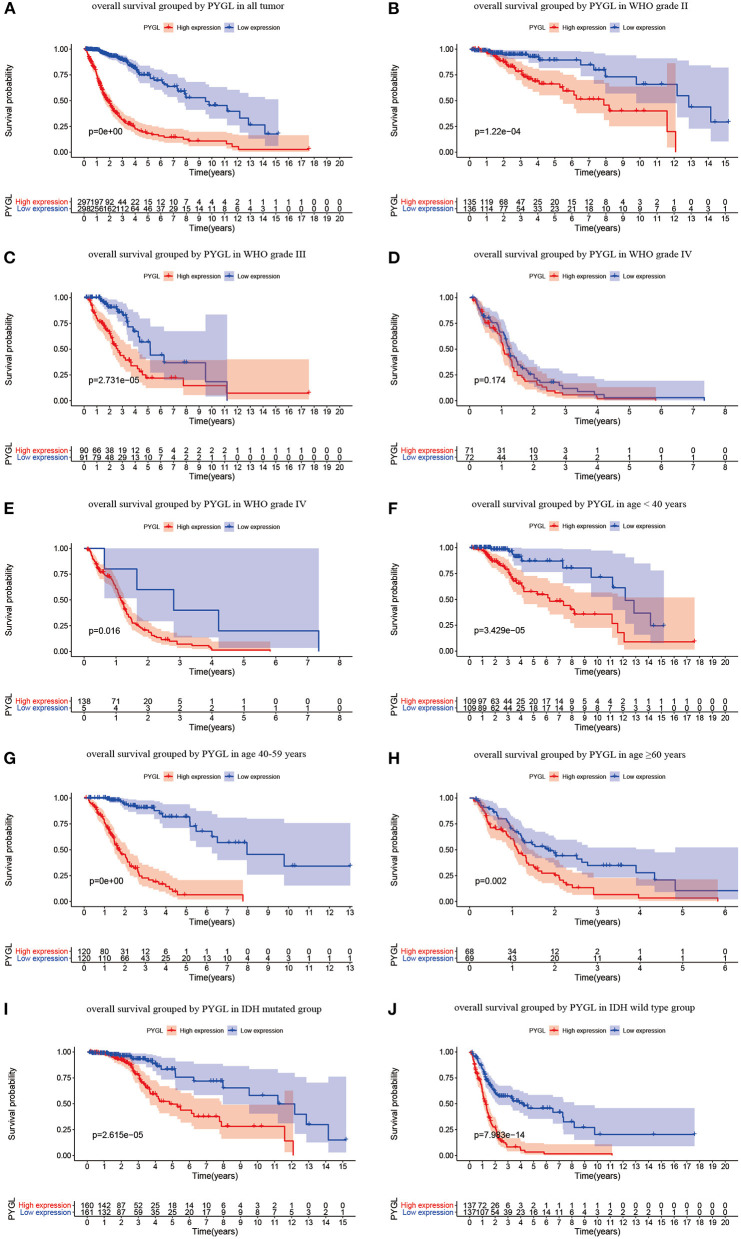
Kaplan-Meier (KM) survival curves for OS in glioma. **(A)** KM curves for OS in glioma for all cases. **(B)** KM curves for OS in gliomas of WHO grade II. **(C)** KM curves for OS in gliomas of WHO grade III. **(D)** KM curves for OS in glioma of WHO grade IV with the median of PYGL expression in gliomas of WHO grade IV as a threshold. **(E)** KM curves for OS in gliomas of WHO grade IV with the median of PYGL expression in all cases as a threshold. **(F)** KM curves for OS in gliomas of age <40 years. **(G)** KM curves for OS in gliomas of age 40–59 years. **(H)** KM curves for OS in gliomas of age ≥60 years. **(I)** KM curves for OS in gliomas with IDH mutation. **(J)** KM curves for OS in gliomas with IDH wild type.

**Figure 3:**
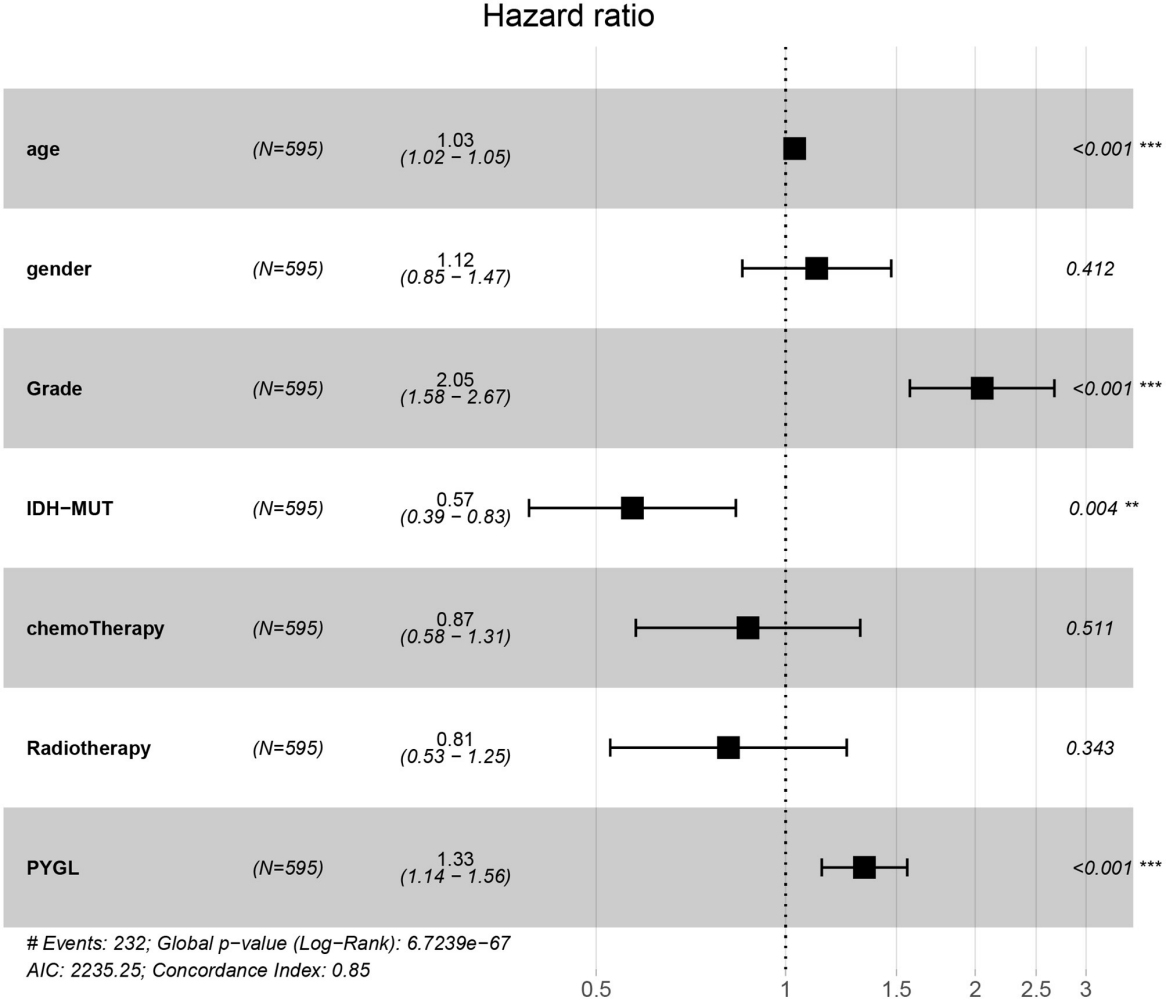
Multivariate Cox hazards regression analyses of age, gender, grade, chemotherapy, radiotherapy and PYGL. (**P < 0.005, ***P < 0.001).

**Figure 4:**
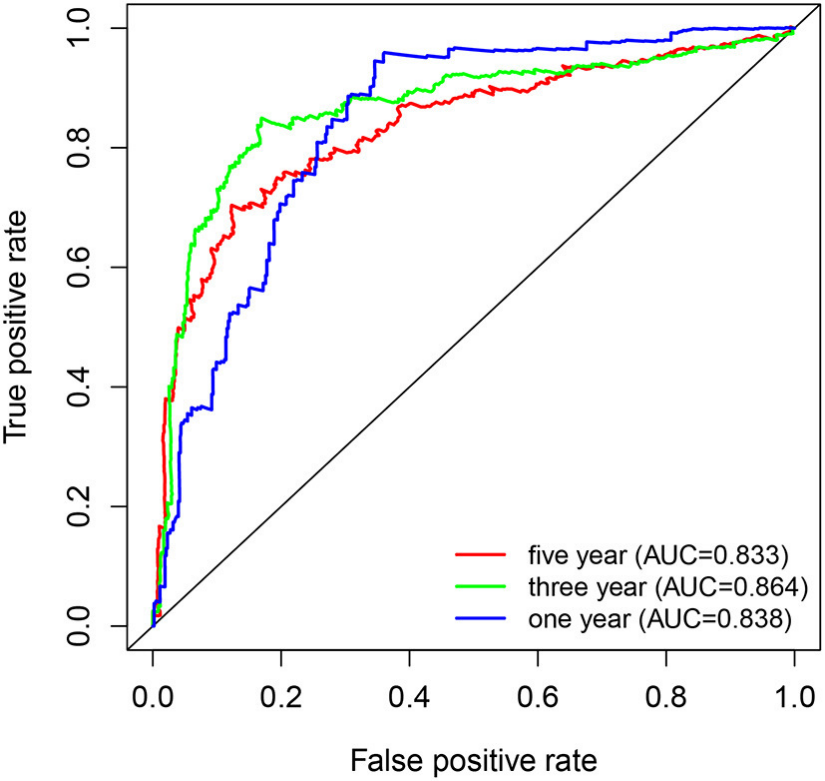
ROC curves of PYGL expression in predicting 1-, 3- and 5-year survival of glioma patients.

Kaplan-Meier survival curves demonstrated that high PYGL expression was associated with poor OS (*P* < 0.05; Figures 2A–C,E–J). Subgroup analysis showed that the high expression of PYGL had a significant effect on the OS of WHO grade II/III and different age groups of gliomas. In the WHO grade IV group, when the threshold was the median of the patients in the group, the high expression of PYGL had no significant effect on OS (*P* = 0.174; Figure 2D); when the threshold was the median of all patients, the high expression of PYGL had a significant effect on OS (*P* = 0.016; Figure 2E). Univariate and multivariate Cox hazards regression analysis indicated that PYGL expression was an independent risk factor for glioma patients (hazard ratio [HR] = 1.43, 95% confidence interval [CI]: 1.23–1.66, *P* = 4.30e-06; Table 2; Figure 3). The AUC values were 0.838 (1-year ROC), 0.864 (3-year ROC), and 0.833 (5-year ROC), which showed that the expression of PYGL could reliably predict the 1-, 3- and 5-year survival rate of patients with gliomas (Figure 4). The C index was 0.81, indicating that high PYGL expression had medium accuracy in predicting poor prognosis in human gliomas. Overall, these results indicated that the expression of PYGL had independent predictive value in the prognosis of glioma patients and high PYGL expression predicted poor prognosis in human gliomas.

**Table 2:** Univariate analysis and multivariate analysis of the correlation between PYGL expression with OS among glioma patients.

**Parameter**	**Univariate analysis**			**Multivariate analysis**		
	**HR**	**95% CI**	***p*-value**	**HR**	**95% CI**	***p*-value**
Age	1.07	1.06–1.08	**2.44e–36**	1.04	1.02–1.05	**1.24e–09**
Gender	1.27	0.98–1.66	0.07	1.13	0.86–1.48	0.38
Grade	4.08	3.39–4.91	**1.54e–49**	2.30	1.78–2.98	**3.06e–10**
IDH mutation	0.21	0.15–0.28	**2.30e–26**	0.57	0.36–0.83	**0.0037**
Chemotherapy	2.21	1.57–3.11	**5.36e–06**	0.88	0.58–1.32	0.54
Radiotherapy	2.30	1.59–3.32	**1.02e–05**	0.83	0.54–1.28	0.40
PYGL	2.29	2.05–2.56	**3.69e–48**	1.43	1.23–1.66	**4.30e–06**

*Bold values indicate P < 0.05. HR, hazard ratio; CI, confidence interval*.

### PYGL-Related Signaling Pathway

**Figure 5:**
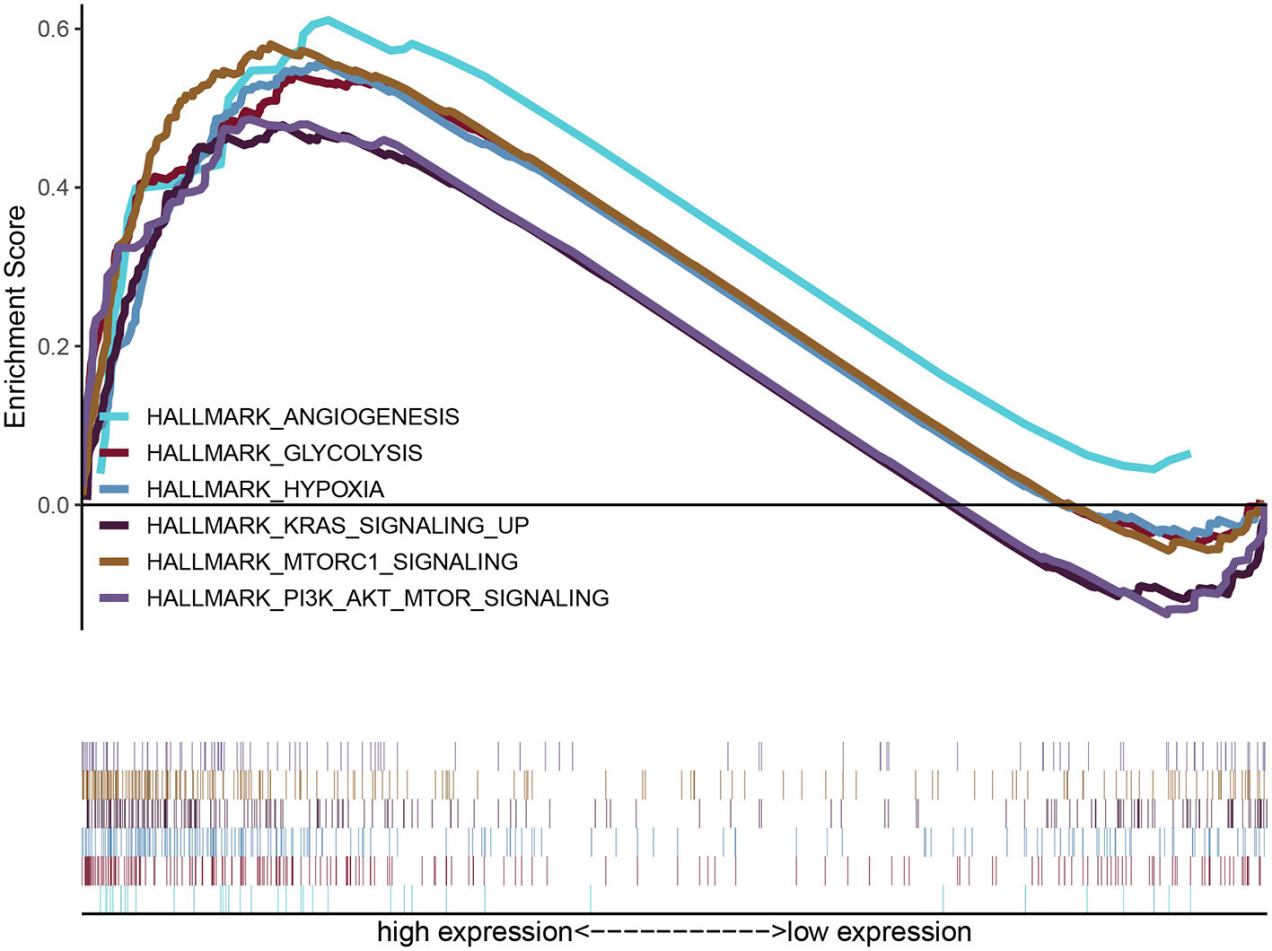
Enrichment plots from GSEA of PYGL. GSEA results showing differential enrichment of genes related to MTORC1 signaling, glycolysis, hypoxia, PI3K/AKT/mTOR signaling, KRAS signaling up and angiogenesis in glioma cases with high PYGL expression.

GSEA uncovered the relationship between the expression of PYGL and hallmark gene sets (Table 3, Figure 5). Gene sets related to MTORC1 signaling, glycolysis, hypoxia, PI3K/AKT/mTOR signaling, KRAS signaling up and angiogenesis were differentially enriched in the high PYGL expression phenotype. However, there was no gene set differentially enriched with the low PYGL expression phenotype.

**Table 3:** Gene sets enriched in the high PYGL expression phenotype.

**Gene set name**	**NES**	**NOM *p*-value**	**FDR *q*-value**
HALLMARK_MTORC1_SIGNALING	1.990583	0.005725	0.006979
HALLMARK_GLYCOLYSIS	1.983647	0	0.006703
HALLMARK_HYPOXIA	1.942395	0.005747	0.006133
HALLMARK_PI3K_AKT_MTOR_SIGNALING	1.820291	0.001919	0.017528
HALLMARK_KRAS_SIGNALING_UP	1.766303	0.018634	0.023621
HALLMARK_ANGIOGENESIS	1.758201	0.008016	0.022896

### PYGL Was Highly Expressed in Immune Cells and Malignant Cells

**Figure 6:**
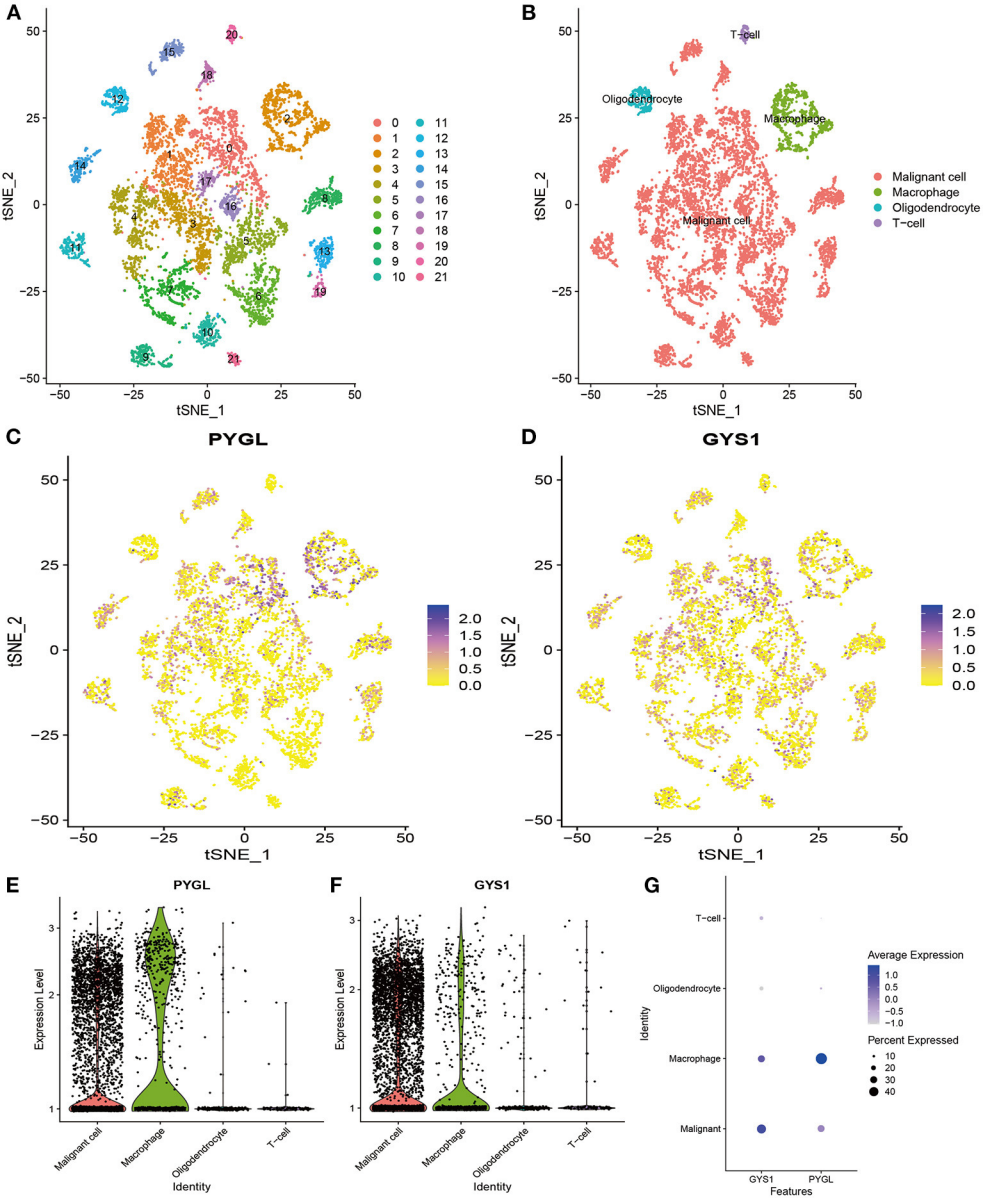
Analysis of Single-cell mRNA sequencing. **(A)** Two-dimensional TSNE plot depicted that 7,930 single cells were classified into 22 clusters and shown with distinct colors. **(B)** Twenty-two clusters were divided into 4 types of cells: malignant cell, macrophage, oligodendrocyte and T-cell. **(C)** The expression of PYGL in each cell. **(D)** The expression of GYS1 in each cell. **(E)** The violin plot showed the expression of PYGL in each type of cell. **(F)** The violin plot showed the expression of GYS1 in each type of cell. **(G)** The bubble plot showed the expression of PYGL and GYS1 in each type of cell.

Since PYGL has the greatest influence on GBM, we further studied the distribution of high expression of PYGL in different types of GBM cells by analyzing single-cell RNA sequencing data. 7,930 GBM cells were classified into 22 clusters and finally divided into four types of cells according to the mark gene (10): malignant cell, macrophage, oligodendrocyte and T-cell (Figures 6A,B). Violin plots, scatter plots and bubble plots showed that PYGL and GYS1 were expressed more highly in macrophage and malignant cells than in oligodendrocytes and T-cells (Figures 6C–G). In summary, these results indicated that glycogen synthesis and breakdown were active in tumor-associated macrophages (TAMs) and malignant cells.

## Discussion

In this study, we found that the expression level of PYGL was positively correlated with the malignant grade of gliomas. Although there was also a positive correlation between age and PYGL expression, the correlation analysis showed that the correlation between malignant grade and age was very low. This indicated that the high expression level of PYGL in high-grade gliomas was not caused by the age of the patient. We also found that the expression level of PYGL was negatively correlated with the survival time of patients with gliomas. In WHO grade IV glioma groups, by adjusting to a lower threshold and re-conducting survival analysis, the results showed that the effect of survival time was significant, which proved that the high expression of PYGL was related to the worse prognosis of patients with WHO grade IV gliomas. Therefore, the high expression of PYGL has independent predictive value in the poor prognosis of glioma patients.

PYGL was included in a list of the 99 genes included in the hypoxia “metagene,” which has been proved to be used for predicting the poor prognostic outcome both in HNSCC and breast cancer. Recently, a weighted gene co-expression network analysis (WGCNA) study suggests that PYGL protein can be used as the prognostic biomarker for the survival of pancreatic ductal adenocarcinoma (11). Meanwhile, there have been two other isoforms of PYGL [e.g., PYGM were remarkably under-expressed in HNSCC and PYGB was over-expressed in hepatocellular carcinoma tissues, separately (12, 13)] also exist in human cells. However, experimental data of bevacizumab treatment suggest that, unlike PYGL, neither PYGM or PYGB exhibited a detectable increase in U87 (glioblastoma) cells (6). Thus, we focus on PYGL only to explain the malignancy of glioma in this study.

Despite the high expression of PYGL is related to the malignant proliferation of brain cancer cells, the underlying mechanism of it is still unclear (14). Hypoxia is a common feature of solid cancer, which is caused by the mismatch between cell oxygen supply and oxygen consumption (15). Previous studies have found that PYGL levels increased more slowly in hypoxia, and reached maximal levels at 72 h, and analysis of PYGL expression in tumor cells revealed distinct patterns of induction by hypoxia (0.1% O_2_) that were consistent at both the mRNA and protein level (6). It has been reported that glycogen metabolism is upregulated in tumors *in vivo* and cancer cells *in vitro* in response to hypoxia. To our knowledge, glycogen metabolism is a key pathway induced by hypoxia, necessary for optimal glucose utilization, which represents a targetable mechanism of metabolic adaptation. Under the condition of glycogen metabolism, glycogen accumulated in the tumor is decomposed into glucose-1-phosphate (G1P) by PYGL, then transformed by the enzyme phosphoglucomutase (PGM) into glucose-6-phosphate (G6P), and enter the pentose phosphate pathway (PPP) to produce a large amount of reducing cofactors (NADPH) (16). Although NADPH can scavenge the reactive oxygen species (ROS) that led to p53-dependent aging and weakens the proliferation of tumors (17), a growing body of evidence has demonstrated that ROS significantly promotes the proliferation of tumor cells (18). In addition, many observations have found that glycogen accumulation markedly occurred in U87 cells under hypoxia (6). Therefore, we can presume that PYGL was likely to be involved in the main glycogen phosphorylase (GP) activity required for glycogen breakdown in hypoxic cells, and helps to promote further cell proliferation in the tumor.

In this study, the result of GSEA indicated that the high expression of PYGL may be related to hypoxia, glycolysis, and some pathways of tumorigenesis and proliferation. Our enrichment plots data suggest that PYGL may induce glioma proliferation through up-regulation of mTORC1 signaling, PI3K/AKT/mTOR signaling, KRAS signaling up, and angiogenesis pathway. To our knowledge, mTORC1 is one of the distinct multi-protein complexes of the functional kinase mTOR, which consisted of the core subunits and regulatory-associated protein of mTOR, 40kDa Proline-rich AKT substrate (PRAS40), and DEP domain-containing mTOR-interacting protein (DEPTOR) (19). It can not only stimulate the biosynthesis of macromolecules: proteins, lipids, and nucleic acids, but also can promote the production of energy (ATP), NAPDH, and certain macromolecule precursors required for biosynthesis (20). At this point, we presumed that PYGL activates the central node of mTORC1 and alternates the glycogen metabolism by reprogrammed the glycolytic pathway, and then allows glioma cells to survive and adapt to restricted nutrition conditions (21). PI3K, which functions as a heterodimer of catalytic and regulatory subunits, is another protein kinase complex. Previous studies have found that PI3K generates PIP3 which specifically binds AKT and PDK1 promoting the phosphorylation and activation of AKT by PDK1. And AKT activates and stimulates mTORC1 kinase activity via relieving the inhibition of Rheb GTPases (22, 23). Cumulatively, PYGL may involve the integration of signal transduction mechanisms through PI3K signaling to the activation of mTORC1 signaling. Moreover, it is well-accepted that KRAS is the most commonly mutated RAS oncogene isoform, and such mutation will constitutively activate thereby enhancing downstream signaling and leading to tumorigenesis (24). In the past, evidence-based observation suggested that interaction between mutated RAS cells and PI3K, and the maximal PI3K signaling is essential for the initiate tumor formation (25, 26). Although KRAS-mutant signaling plays an important role in tumorigenesis, only signaling through the PI3K/AKT pathway seems to be necessary for the maintenance of tumor growth (27–29). Furthermore, it had been proven that PI3K regulates tumor growth and angiogenesis by activating AKT and other targets, and by inducing HIF-1 and VEGF expression. And angiogenesis is required for tumor growth and metastasis (30). Therefore, we can hypothesize that PYGL involves in the crosstalk of these signaling molecules and cellular events during tumor growth, metastasis, and angiogenesis (30). Besides, it is glucose metabolism that has been regulating the cell cycle and apoptosis of glioma cells (31).

Notably, our single-cell analysis data showed that not only malignant cells but also macrophage expressed PYGL and GYS1 highly. Such results are consistent with previous studies that hypoxia induced an increase in the expression of GYS1, and the liver isoform of PYGL in glioblastoma cells (6). Studies of tumors showed that GYS1—the most important rate-limiting enzyme functioning in the last step of glycogen synthesis, was rapidly induced under hypoxic conditions and positively correlated with glycogen accumulation in glioblastoma (21, 32). At this point, our finding further revealed the direct effects of the aberrant glycogen synthesis and decomposition play an interactive role in the proliferation of glioma. Although previous studies have demonstrated that heightened expression of G6P can potentially serve as a novel therapeutic target that renders cancer cells less aggressive, our findings suggest that the additional interaction of glycogen metabolism has gone beyond its role as a source of energy-supplying to oncogenesis (21, 33).

More interestingly, we found for the first time that glycogen synthesis and decomposition are enhanced in the glioblastoma TAMs, while relatively rare in the T and oligodendrocyte cells. Previous histologic analysis of malignant glioma reveals TAMs composed of microglia, monocytes/macrophages, astrocytes, endothelial cells, neural stem/progenitor cells, pericytes, and other immune cell infiltrates (34). Under pathological conditions, peripheral monocytes can enter the CNS from the blood through a disrupted blood brain barrier, and further showing a tendency to richer than oligodendrocytes (35, 36). To our best knowledge, glioma TAMs can come from tissue-resident macrophages or macrophages recruited from blood, and these macrophages account for 30–50% of the tumor mass (37, 38). There is evidence to suggest that macrophages can use glycolysis-derived G6P which is channeled through the PPP to produce large amounts of NADPH required for macrophage-mediated inflammatory responses (39). Therefore, we innovatively put forward hypotheses that gliomas are rich in PYGL-mediated G6P which may participate in tumor growth, and glycogen metabolism is an important event in the tumor immune escape process (40).

## Limitation

Our study accounts for a number of limitations that we would like to highlight. The major limitation is the lack of *in vitro* or *in vivo* models to validate our findings. All data were collected retrospectively from the analysis of data from public databases (TCGA and GEO) and thus the results haven't been clinically validated outside these. In this study, research data varied among participating units, leading to data heterogeneity. There was no explicit discussion of the difference in PYGL expression between glioma and normal brain. In addition, there has been no data reported regarding the extent of surgical resection and/or residual tumor volume in the TCGA and GEO databases. Further prospective experimental validation is required to investigate the relationship between the key metabolic gene in the oxidative decarboxylation of isocitrate in the tricarboxylic acid cycle and the PYGL expression.

## Conclusion

In summary, this study has shown that the high expression of PYGL has independent predictive value in the poor prognosis of patients with gliomas. TAMs express PYGL and GYS1 highly, which may be one reason for glioma proliferation and immune escape.

## Data Availability Statement

All datasets presented in this study are included in the article/supplementary material.

## Author Contributions

C-yZ, C-hH, and C-hL contributed to data acquisition. C-yZ, C-hL, and R-zZ performed the statistical analysis and prepared the manuscript. C-yZ and R-zZ drafted this manuscript. X-yL supervised the study. All authors read and approved the final manuscript.

## Conflict of Interest

The authors declare that the research was conducted in the absence of any commercial or financial relationships that could be construed as a potential conflict of interest.
